# Anti-Inflammatory Potential of Brassicaceae-Derived Phytochemicals: In Vitro and In Vivo Evidence for a Putative Role in the Prevention and Treatment of IBD

**DOI:** 10.3390/nu15010031

**Published:** 2022-12-21

**Authors:** Adele Cicio, Rosa Serio, Maria Grazia Zizzo

**Affiliations:** 1 Department of Biological, Chemical and Pharmaceutical Sciences and Technologies (STEBICEF), University of Palermo, Viale delle Scienze, ed 16, 90128 Palermo, Italy; 2 ATeN (Advanced Technologies Network) Center, Viale delle Scienze, University of Palermo, 90128 Palermo, Italy

**Keywords:** Brassicacae, inflammation, inflammatory bowel disease

## Abstract

Inflammatory bowel disease (IBD) is a group of intestinal disorders, of unknown etiology, characterized by chronic inflammation within the gut. They are gradually becoming critical because of the increasing incidence worldwide and improved diagnosis. Due to the important side effects observed during conventional therapy, natural bioactive components are now under intense investigation for the prevention and treatment of chronic illnesses. The Brassicaceae family comprises vegetables widely consumed all over the world. In recent decades, a growing body of literature has reported that extracts from the Brassicaceae family and their purified constituents have anti-inflammatory properties, which has generated interest from both the scientific community and clinicians. In this review, data from the literature are scrutinized and concisely presented demonstrating that Brassicaceae may have anti-IBD potential. The excellent biological activities of Brassicacea are widely attributable to their ability to regulate the levels of inflammatory and oxidant mediators, as well as their capacity for immunomodulatory regulation, maintenance of intestinal barrier integrity and intestinal flora balance. Possible future applications of bioactive-derived compounds from Brassicaceae for promoting intestinal health should be investigated.

## 1. Introduction

Inflammatory bowel disease (IBD) includes a set of intestinal disorders mainly rep-resented by Ulcerative colitis (UC) and Crohn’s disease (CD), induced by chronic inflammation within the gastrointestinal tract [1,2,3].

UC and CD differ for symptomatology, treatment and part of the gut affected. UC is characterized by a superficial inflammation with erosin ulcerations involving mainly colonic tract until-rectum. UC presents bloody diarrhea with mucinous stool [4]. In contrast, in CD any part of gut can be affected with a transmural and discontinued inflammation, fibrosis fistula and strictures leading abdominal pain and obstruction or diarrhea [5]. So far, the etiology of both CD and UC is unknown, although pivotal roles seem to be played by factors such as genetic susceptibility, environment, intestinal microbiota, and, mainly, a worsening and inappropriate mucosal immune response against luminal antigens leading to the development of intestinal inflammation [3,6,7]. An active cross talk between immune and nonimmune cells is responsible for tissue damage and T cells play a central role increasing the amount of pro-inflammatory cytokines, influencing the disease progression, [8]. IBD-related mucosal inflammation starts to develop in predisposed individuals, as a result of an inflated mucosal immune response directed against luminal antigens. In detail, inflammation is enhanced at intestinal level by nuclear factor-κB (NF-κB) activation, production of pro-inflammatory mediators, as cytokines (tumor necrosis factor alpha (TNF-α), interleukin (IL) 6 (IL-6) and interleukin 1 beta (IL-1β) and inflammatory mediators, as myeloperoxidase (MPO), cycloxygenase-2 (COX-2) and inducible nitric oxide synthase (iNOS), together with a concomitant reduction in antioxidant levels [9,10,11].

IBD incidence and prevalence are high worldwide; so far, most studies are related to North America and Europe where prevalence exceeds 0.3% [12], although in the last few years the body of work is gradually increasing in Asia and Latin America in parallel with the continuous progress of global industrialization, urbanization and improved diagnosis [12]. According to these statistics, IBD has become a concerning public health issue and a common cause of gastrointestinal morbidity [13,14]. Moreover, although rarely fatal, IBD symptomatology (pain, vomiting, diarrhea) greatly diminish the quality of life and increasing evidence indicates that IBD can increase the risk of serious complications [15].

Current medical IBD therapies, as corticosteroids, aminosalicylates, immunomodulators and biological agents, aim to modulate the inflammatory response. However, the treatment effect for many patients is not obvious, not providing complete and life-long relief, and having various side effects [16,17,18]. Therefore, it is mandatory to research new therapies with minimal side effects. Increasing evidence suggests the use of complementary, alternative therapies, may have efficacy in treating IBD, and research is moving on the identification of dietary compounds with anti-inflammatory actions. Moreover, the use of drugs of natural origin is growing due to the low cost and to the lack or reduced side effects compared to the conventional drugs [19,20]. Several recent reports document the use of natural phytochemicals, with anti-inflammatory and antioxidant activity, as flavonoids and phenolic compounds, derived from Plant/macrofungi/microalgae, which via modulation of several inflammatory mediators interfere with the biochemical and molecular inflammatory pathways associated to IBD [21,22,23,24,25].

Brassicaceae, often called Cruciferae or mustard family, are a monophyletic group of about 338 genera and some 3709 species distributed all over the world except Antarctica [26]. A wild ancestor was originally found growing along the Mediterranean; nowadays, a wide range of crops and multiple domestications of these plants have occurred in North America, Mediterranean Europe and Asia [27]. In the human diet, Brassicaceae are consumed as fresh and preserved vegetables, vegetable oils and condiments [28,29]. In addition to their culinary use, Brassicaceae have been extensively used in traditional medicine from ancient times, for instance for the relieve of gastrointestinal disorder symptoms [30]. Therefore, Brassicaceae vegetables are recognized as functional food and dietary supplements; extracts containing Brassicaceae or single compounds isolated from these vegetables are already available [31]. Most of the members of the Brassicaceae family give relevant beneficial effects for human health [32]. Consumption of cauliflower, Brussels sprout*, Brassica oleracea* (kale), green mustard, cabbage, and broccoli are associated to a decreased risk of several types of cancer of the incidence of cardiovascular disease [32,33]; also *Brassica rapa* L. is used to treat a variety of diseases, such as hepatitis, jaundice, throats and in the prevention of obesity [34,35].

The positive effects of Brassicaceae seem to be related to the high contents of biologically active compounds, such as flavonoids (flavonols and anthocyanins, isorhamnetin, kaempferol and quercetin glycosides) [36]**,** phenylpropanoid derivatives [37], hydroxycinnamic acids [32,38], indole alkaloids [39] and sterol glucosides [39]. Moreover, a group of sulphur and nitrogen-containing compounds called “glucosinolates”, present in all cruciferous vegetables, contributes to the plant’s overall defense mechanism, although the chemistries and relative proportions vary from crop to crop [40,41]. Glucosinolates, in addition to being responsible for most of the Brassicaceae beneficial health effects, can also exert toxicological actions depending upon the hydrolysis products with various biological activity. The hydrolysis products as goitrin, thiocyanate ion and several nitriles exert toxic effects [42,43]. Whilst the products hydrolyzed by the enzyme myrosinase, as several isothiocyanates (ITCs), are considered to be responsible of most of the Brassicaceae beneficial health effects [44,45,46] as well as the products of hydrolysis of aliphatic glucosinolates as glucobrassicin (precursor of indole-3-carbinol (I3C), that usually dimerizes into the condensation product 3,3-diindolylme-thane (DIM)) and the glucosinolate glucoraphanin, which is hydrolyzed in sulforaphane (SFN) [47,48]. The beneficial or toxic effects of glucosinates and derivates are dose-dependent and the physiological range remains to be defined. The quality and quantity of glucosinolates as well as of other phytochemicals biosynthesized by Brassicaceae are affected by genetic characteristics and several physical and chemical environmental factors (as harvest time, osmotic and hydric stress, nutrition, photoperiod, relative humidity, seasonality, soil-properties such salinity, pH, chemical composition, toxins, and the temperature) [49,50,51,52]. For instance, Brassicaceae, especially the genus *Brassica*, spontaneously growing in Sicily, home of biodiversity, counts more than ten endemic species; a first characterization of antioxidants and unsaturated fatty acid contents in seeds [53] suggested an high nutritional value and support the idea of further studies analyzing the correlation between the amount of beneficial compounds and the environmental factors of the point of growth. So far, in our laboratory extracts from flowers, fruits and leaves from each endemic Sicilian species were obtained and preliminary data indicate no in vitro toxicity on Caco-2 cells. In addition, cruciferous vegetables have nutritional value due to their high protein content, low fat, vitamins, fibers and minerals, as well as to their low caloric count. Due to the high level of vitamins, Brassicaceae crops have the potential to prevent and remedy malignant and degenerative diseases and, due to the presence of folate, have the potential to reduce the risk of vascular diseases, cancer and neural tube defects [32]. In this view, the present review aims to summarize and discuss the current knowledge regarding the potential anti-inflammatory activity of Brassicaceae-derived natural compounds and their possible role in the prevention and treatment of IBD. These data could encourage new research on possible application of Brassicaceae derived compounds as an integrative natural product with a potential use in the prophylaxis, management, and treatment of IBD. The present review has been carried out by consulting PubMed, Scopus, Science Direct, and Web of Science databases retrieving the most updated articles about this topic prioritizing articles published from 1997 to 2022. We searched a different combination of keywords including: cruciferous vegetables, Brassica, Brassicaceae broccoli, cabbage, Brussels sprouts, bok choy, glucosinolate, sulforaphane AND/OR inflammatory bowel disease, IBD, inflammation.. Reference lists of articles were also reviewed for additional relevant studies. Articles with the following features were included: (1) original research articles investigating the Brassicaceae effects on inflammation; (2) studies conducted using cell cultures, IBD animal models and 3) full papers only. Articles with the following characteristics were rejected: (1) conference abstracts, letters or commentary, editorial and opinion articles lacking original data; (2) articles written in a language other than English. All articles were carefully analyzed by the authors to assess their weaknesses strengths and the more useful articles were selected for the review purpose. The inclusion of an article was based on consensus by both authors. In total, 116 articles meeting the criteria were included in this review.

## 2. Brassicaceae and Inflammation: Evidence of Anti-Inflammatory and Antioxidant Effects In Vitro

Inflammation, as above reported, leads to the upregulation of a series of enzyme and signaling protein or genes in affected cells and tissues. It is a complex process, regulated by cytokines, such TNF-α, IL-6 and IL-1β networks, and by the inductions of many pro-inflammatory genes, such as iNOS, COX-2 and reactive oxygen species (ROS). When the response is not able to resolve the acute inflammatory process it could evolve into a chronic inflammation, which is characterized by excessive levels of pro-inflammatory mediators, that could mediate tissue injury and lead to malignant cell transformation, as in cancer [54]. In this section, in vitro evidence about anti-inflammatory and antioxidant proprieties of Brassicaceae extracts and bioactive molecules will be presented (Table 1).

### 2.1. Inflammatory Mediators

The inhibition of pro-inflammatory mediator production is thought to be an important target in regulating inflammation [9]. Altogether, results from the various experiments indicate that Brassicaceae can inhibit production of pro-inflammatory cytokines (e.g., IL-1β, IL-6, TNF-α) and increase the levels of anti-inflammatory cytokines (e.g., IL-10). However, the effects of Brassicaceae can be influenced by various aspects such as the specific stimulus, cell type, and experimental model used.

#### 2.1.1. Effects of Brassicaceae Extracts

Brassicaceae extracts can act as potential natural anti-inflammatory agents. Indeed, it has been observed that the fresh plant extract *Iberis amara* (or STW 6) is the main component of the Western herbal medicine Iberogast^®^ (or STW 5) mainly used in IBD treatment. Michael’ group [55] demonstrated the ability to reduce the inflammatory response of the fixed herbal combination product STW 5 and of its main component STW 6 in isolated intestinal preparations from 2,4,6-trinitrobenzenesulfonic acid (TNBS)-rat model of inflammation. Both compounds were able to counteract morphological and contractile damages of the intestinal muscle layer. Such effects have been suggested to be ascribed to the inhibition of the release of the pro-inflammatory mediator, TNF-α, by STW 5 and to the activation of the IL-10 pathway, independently from TNF-α pathway, by STW 6. Interestingly, the main component STW 6 was more effective than the whole product, indicating that Brassicaceae may have therapeutic value as anti-inflammatory drugs [55]. Additionally, radish sprout ethanolic extract has been reported to have an inhibitory effect of on the production of inflammatory mediators (TNF-α, IL-1β, iNOS, COX-2, IL-6, and Monocyte Chemoattractant Protein-1 (MCP-1)) in macrophages with Lipopolysaccharides (LPS)-induced inflammation [56]. Similar anti-inflammatory activity in the RAW 264.7 cell line LPS-stimulated, consisting in reduced production of the pro-inflammatory cytokines TNF-α, IL-6 and IL-1β, as well as iNOS gene and protein expression, was determined for the ethyl acetate fraction of *Brassica oleracea var. Italia* characterized by high phenolic and SFN content [57]. In the same cell line, four extracts of Red, Savoy, Green and Chinese (*Brassica oleracea L. var. capitata*) cabbage varieties due to the high phenolic content were able to reduce NO production [58]. In human peripheral blood mononuclear cells (PBMC), methanolic/water extract from broccoli sprouts during germination, presenting a high content of sinapic acid derivatives, glucosinolates flavonoids and sinapoyl components (SADs) are able to exert anti-inflammatory and antioxidant effects [59].

#### 2.1.2. Effects of Brassicaceae-Derived Phytochemicals

Growing observations in various cellular models suggest that also single biomolecules isolated from in Brassicaceae can exert anti-inflammatory effects reducing, at different levels, the production of pro-inflammatory cytokines. Indeed, recently, in an elegant study, Ruiz-Alcaraz et al. [60] demonstrated using the pure compounds of biomolecules present in Brassica that many of them (as glucosinolates, isothiocyanates, hydroxycinnamic acids, flavonols and anthocyanins) showed high anti-inflammatory activities reducing the production of the pro-inflammatory cytokines (TNF-α, IL-6 and IL-1β) in human macrophage-like cell model under low-degree inflammation [60]. This study highlights the potential utility of these compounds in the prevention or amelioration of chronic inflammatory diseases and encourage studies to identify selected member of Brassicaceae family able to provide the right quota of bioactive compounds to be included in dietary intervention.

Studies demonstrated that pigments from red cabbage (*Brassica oleracea* L. var.) juice with high contents of compound as immune-malvidin glycosides, including malvidin 3-glucoside (oenin), malvidin 5-glucoside and malvidin 3,5-diglucoside, exert anti-inflammatory effects on LPS-stimulated murine splenocyte cultures, modulating the levels of cytokines, with an increase in the anti-inflammatory, as IL-10 and decrease of the pro-inflammatory as IL-6 [61]. Down-regulation of inflammatory mediators, as TNF-α, IL-6 and IL-1β and/or increase in anti-inflammatory mediators, as IL-10, have been demonstrated also for other components isolated from Brassicaceae, as for Arvelexin isolated from *Brassica rapa*, in RAW 297.4 cells [62]**.**

### 2.2. Inflammatory Pathways

The nuclear transcription factor, NF-κB, is a major regulatory component of the inflammatory response. The expression of iNOS and the COX-2 genes depends upon the binding of NF-κB to the promoter regions [63]. NF-κB plays a role also in the expression of various pro-inflammatory genes, as cytokines, chemokines, and adhesion molecules. In unstimulated cells, NF-κB dimers are the inactive form in the cytosol due to the interaction with inhibitor of kappa B (IκB) proteins. Upon selective stimulation by a wide variety of proinflammatory stimuli, IκB proteins are phosphorylated by the IκB kinase (IKK) complex leading to nuclear translocation of NF-κB, which, in turn, promotes the target gene transcription [64]. Therefore, NF-κB is an attractive target to manage inflammation-related diseases.

#### 2.2.1. Effects of Brassicaceae Extracts

Water extract of Bok Choy (*Brassica campestris var. chinensis*) Sprouts, a representative Brassicaceae crop and consumed worldwide, is able to exert immunomodulatory effects through Toll-like receptor (TLR2)-dependent activation of c-Jun N-terminal kinase (JNK), NF-κB and Protein kinase B (PKB, or Akt) signalling in RAW 264.7 [65]. In addition, *Brassica napus* L. hydrosols, targeting NF-κB pathway, exerts anti-inflammatory effects decreasing the generation of NO and prostaglandin (PG) E in LPS-stimulated RAW 264.7 cells [66].

Moreover, the water and methanolic extracts from *Brassica oleracea L. convar*. *acephala* var. *sabellica, containing mainly* flavonols, as quercetin and kaempferol hydroxycinnamates (chlorogenic, caffeic, ferulic and p-coumaric acid) had an influence on the adhesion of neutrophils, in TNF-α-stimulated endothelial cells and the expression of various cell adhesion molecules [67].

#### 2.2.2. Effects of Brassicaceae-Derived Phytochemicals

A variety of Brassicaceae-derived phytochemicals seem to act as NF-κB inhibitors leading to down-regulation of inflammatory mediators. For instance, phytochemicals contained in watercress as SFN, phenethyl-isothiocyanate (PEITC), 8-methylsulphinyloctyl isothiocyanate (MSO) and indole-3-carbinol (I3C) can downregulate activation of NF-κB induced by LPS and suppress COX-2, iNOS, and prostaglandins expression in cultured mouse macrophages [68,69]. As above reported, Arvelexin from *Brassica rapa* exerts anti-inflammatory effects in LPS stimulated RAW264.7 preventing NF-κB activation and down-regulating TNF-a, IL-6 and IL-1β, iNOS and COX-2, gene expression [62].

### 2.3. Antioxidant Effects

In addition to anti-inflammatory potential, Brassicaceae can also present antioxidant potential. Numerous in vitro assays, such as: 2,2-Diphenyl-1-Picrylhydrazyl (DPPH) radical scavenging capacity assay [36,59,70,71,72,73,74,75,76,77,78]; 2-20-azino-bis (3-ethylbenzothiazoline-6-sulphonic acid) diammonium salt (ABTS)-radical scavenging capacity assay; Ferric-reducing antioxidant power assay (FRAP) [58,79,80,81] or oxygen radical absorbance capacity (ORAC) assay [82,83] demonstrated that extracts from various components of the Brassicaceae family, as broccoli, cabbages and cauliflowers have also high antioxidant power. In cellular models, Brassicaceae has been reported to inhibit iNOS expression and function, and thereby NO production [62]. The oxidized metabolite of nitrogen is a well-known toxic agent playing a crucial role in the promotion of several chronic diseases as inflammatory bowel disease, septic and hemorrhagic shock and certain autoimmune disorders. Different extracts act also as ROS scavenger, inhibitors of ROS production, and activators of antioxidant enzymes.

The above mentioned study of Shin’s group also highlighted the ability of Arvelexin from *Brassica rapa* in the inhibition of iNOS and COX2 gene expression in RAW 264.7 cells [62]. A protective effect against protein oxidation by broccoli flower Butanol fraction was related to the ability to scavenge NO as well to the inhibition of lipid peroxidation, and correlated to the high phenol content and to active components, especially glycosides and hydroxycinnamic acid, either in vitro or in vivo model of diabetes [84]. Lastly, carotenoids and polyphenols from plums and cabbages *Brassica oleracea* var. *sabellica*, have shown to reduce inflammation in cellular models of the intestinal epithelium, modulating different important antioxidant enzymes, e.g., catalase, glutathione transferase, and superoxide dismutase (SOD) [85]. Once more, these observations indicate the need to further investigate Brassicaceae as a promising source of phytochemicals for new alternative and complementary therapeutic approaches regarding inflammatory diseases.

**Table 1:** In vitro effects of Brassicaceae on different cellular models.

Brassicaceae	Type of Extract	Dose	Cell Model	Reported Activity	Reference
**BRASSICACEAE EXTRACTS**					
*Iberis amara*	Fresh plant extract dissolved in water or in 31% V/V ethanol solution	STW5 128 μg/mL STW6 6 μg/mL	LPS-stimulated monocytes	↑ IL-10 ↓TNF-α	[55]
*Radish sprout*	Ethanol extract		LPS-stimulated RAW-264.7	↓ TNF-α, IL-1β, IL-6, MCP-1 iNOS, COX-2	[56]
*Broccoli* (*Brassica oleracea var. italia*) *florets*	Methanol extract	25–50–100 μg/mL	LPS-stimulated RAW-264.7	↓ NO, iNOS, TNF-α, IL-1β, IL-6 ↓ IκB-α degradation and NF-κB	[57]
*Brassica oleracea* L. *var. capitata*	Methanol extract	25–50–100 μg/mL	LPS-stimulated RAW-264.7	↓ NO production	[58]
*Brassica oleracea* L. convar. *Botrytis* var. *cymosa* 6-day-sprouts	Methanol/water extract	75–100 μg/mL	Human PBMC	↓ TNF-α, IL-6 and IL-1β ↑ IL-10 ↓ NO, iNOS, COX-2, PGE_2_	[59]
*Bok Choy* (*Brassica* *campestris var. chinensis*) *Sprouts*	Water extract	50–100 μg/mL	LPS-stimulated RAW-264.7	↓ NO, iNOS, IL-1β, IL-6 and TNF-α ↓ MAPK activation	[65]
*Brassica napus* L.	hydrosols	1–2.5–5 %	LPS-stimulated RAW-264.7	↓ NO, iNOS, COX-2, PGE_2_, NF-κB	[66]
*Brassica oleracea* L. *Convar. acephala* Var. sabellica	Methanolic extracts		TNF-α- stimulated HUVEC	↓ E-selectin, VCAM-1 and ICAM-1	[67]
**BRASSICACAE-DERIVED PHYTOCHEMICALS**					
*Brassica*	Glucosinates and flavonoids	25–50 μM	human macrophage-like	↓ TNF-α, IL-1β, IL-6	[60]
*Brassica oleracea. var. capitate*	Pigments from juice	20–100–500 μg/mL	LPS-stimulated murine splenocyte	↑ IL-10 ↓ IL-6	[61]
*Brassica rapa*	Arvelexin	25–50–100 μM	LPS-stimulated RAW-264.7	↓ NF-κB activation ↓ TNF-α, IL-6 and IL-1β ↑ IL-10 ↓ NO, iNOS, COX-2, PGE_2_	[62]
*Watercress* *Nasturtium* *officinalis*	PEITC and MSO	1–5–10 μM	LPS-stimulated RAW-264.7	↓ Metalloproteinase-9 activity and invasiveness	[68]
*Brassica*	SFN	25–50–100 μM	LPS-stimulated RAW-264.7	↓ NO, iNOS, TNF-α, COX-2, PGE_2_	[69]
*Plum cabbage* (*Kale, Brassica oleracea var. sabellica*)	Carotenoids and polyphenols	Digesta (500 mg of dried matrix and 3 g cream) diluted 1:8 with medium	LPS-stimulated Caco-2 LPS-stimulated Caco-2/HT-29-MTX, and THP-1	↓ Catalase, glutathione transferase, and SOD	[85]

Abbreviations: COX-2: cyclooxygenase-2. HVCEC: cultured human vena cava. ICAM-1: intercellular adhesion molecule-1. IL: interleukin. iNOS: inducible nitric oxide synthase. ITCs: isothiocyanates. LPS: bacterial lipopolysaccharide. MCP-1: Monocyte Chemoattractant Protein-1. MSO: 8-methylsulphinyloctyl isothiocyanates. NF-Κb: nuclear factor-κB. NO: nitric oxide. PBMC: peripheral blood mononuclear cells. PEITC: Beta-phenylethyl isothiocyanate. PGE-2: prostaglandin E2. SFN: sulforaphane. SOD: superoxide dismutase. TNF-α: tumor necrosis factor alpha. VCAM-1 vascular cell adhesion molecule-1. ↓ decrease. ↑ increase.

## 3. Brassicaceae and Inflammation: Evidence of Anti-Inflammatory and Antioxidant Effects In Vivo Focusing on IBD 

During the past decade, in various animal models the effects of Brassicaceae extracts and components have been tested, highlighting anti-inflammatory, anti-oxidant, anti-septic and anti-carcinogenic effects. Due to the multifactorial feature of IBD, different animal models have been established to investigate potential beneficial effects of various therapeutic strategies and the underlying mechanisms [86,87,88].

This section will summarize the results from in vivo studies regarding Brassicaceae regulation of inflammatory processes, focusing the attention on IBD models (Table 2).

### 3.1. Effects of Brassicaceae Extracts

Oral administration of *Brassica oleracea* var. *capitata rubra* extract, rich in anthocyanins, attenuates experimental colitis in acute and chronic mouse models of IBD, inducing both curative and prophylactic effects. A significant decrease in expression of IL-1β and TNF-α was observed, as well as a reduction in MPO level and an improvement of macroscopic and microscopic score of inflammations [89]. Interestingly, the anti-inflammatory effect of extract was independent of the antioxidant effects attributed to its components. Whether or not there was an impact on gut microbioma was not solved. In addition, Brassicaceae *Raphanus sativus* L. (RSL) seed water extract, has been demonstrated to exhibit anti-oxidant, anti-inflammatory, and anti-septic actions in experimental TNBS- or dextran sodium sulfate (DSS)- induced colitis in rats. Oral administration of RSL seed water extract suppressed intestinal inflammatory damages in both animal models, decreasing the MPO activity and the secretion of TNF-α and IL-1β, inhibiting also malondialdehyde production and glutathione reduction in the colon of colitis rats. A reduction in monocyte chemotactic protein-1, iNOS, and intercellular adhesion molecule-1 was observed too. The beneficial actions of such an extract seems to be related to the modulation of NF-κB-mediated pathways [90].

Subsequently, nanoparticles isolated from broccoli extracts (BDNs) have been demonstrated to play a role in prevention of experimental colitis in mouse, [91]. Oral administration of BDNs protect mice with DSS-induced colitis, preventing DSS-induced weight loss, reducing inflammatory infiltrate in the mucosa, affecting colonic goblet cells, and significantly improving colonic shortening. Moreover, BDN treatment induced activation of adenosine monophosphate-activated protein kinase (*AMPK*) and reduced the mRNA levels of TNF-α, IL-17A, and *Interferon gamma* (*IFN*-*γ*) and increased the expression of IL-10. Beneficial effects of BDNs were also observed in a model of colitis induced by the adoptive transfer of naive T cells into *Rag*1-deficient mice. BDN oral treatment decreased the main inflammatory marker levels and significantly reduced number of CD4^+^ T cells in mesenteric lymph nodes. BDNs effects were due to activation of the enzyme adenosine monophosphate activated protein kinase which is involved in the immune homeostasis process in dendritic cells [91].

Ethanol extract of the Brassicaceae *Wasabia Japonica* prevented the development of colitis in DSS mouse model through inhibition of the NF-kB signaling pathway and recovery of epithelial tight junctions [92].

The Brassicaceae plant *Camelina sativa,* native mainly in European countries, has been investigated due to its high content of n-3 fatty acids, flavonoids and glucosinolates [93]. Recently, it has been shown that acute administration of *Camelina sativa* defatted seed meal in 2,4-dinitrobenzenesulfonic acid (DNBS)-treated rats counteracted the persistence of visceral hyperalgesia by reducing the intestinal inflammatory damage and preventing enteric neuron damage via activation of peroxisome proliferator-activated receptor alpha (PPAR-α) receptors [94]. Once more, investigation of the effects exerted by single active constituents of *Camelina sativa* is worthwhile.

The same research group demonstrated that the water extract of Brassicaceae plant *Eruca sativa* Mill, displays visceral anti-nociceptive effect in a DNBS- rat model. This effect, attributable to the high glucosinolate content, was suggested to be due to the release of hydrogen sulphide (H_2_S) and the positive modulation of Kv_7_ potassium channel activity [95]. The Brassicaceae Maca (*Lepidium meyenii*) is the only source of Macamides, a class of bioactive amide alkaloids. Crude extract of Maca has been reported to possess various biological activities, such as antioxidative activity and immune regulation [96,97,98]. Zha et al. [98] identified in maca extract a minor macamide, N-benzyl docosahexaenamide (NB-DHA), with the highest degree of unsaturation among the other macamides and showed that it has protective effects on the DSS-induced in mice, counteracting the weight loss, shortening of colon length, and reducing occult blood occurrence. Moreover, NB-DHA decreased the infiltration of inflammatory cells and levels of pro-inflammatory factors, such as TNF-α, IL-1β, IL-6, and MPO, whereas it increased the level of the anti-inflammatory factor IL-10. Furthermore, NB-DHA showed protective effects on the intestinal epithelial barrier reverting the DSS- induced decreased expression of intestinal tight junction proteins. Authors concluded that NB-DHA may be considered as a promising candidate for the treatment of UC. Targeting NF-κB pathway, via inhibition of the phosphorylation of IκB with a concomitant inhibition of pro-inflammatory cytokine production, has been reported for the anti-inflammatory effects induce by radish sprout ethanolic extract improving macroscopic and microscopic score of colitis in DSS-induced colitis model [56]. Interestingly, radish sprout restored gut microbiota in DSS animals. Authors identified three main hydroxycinnamic acids in radish sprout, showing that at least 1,2-O-disinapoyl glucoside was associated to nitric oxide inhibition and the anti-inflammatory properties [56]. However, further studies are required to link each hydroxycinnamic acid to the production of inflammatory cytokines in either cells or animals. An early study from Lima et al. [99] showed that the vegetable Kale (*Brassica oleracea*), containing minerals, carotenoids, and vitamins (B and C) has a moderate effect on TNBS-induced colitis in rats. The research emphasized that a mixture of phytochemicals in fruits and vegetables through overlapping or complementary effects is more effective that a single phytochemical. In fact, Lima and his group compared the effects of papaya (rich in carotenoids) or *Kale (Brassica oleracea)* administered separately or in combination (40% of papaya and 60% of kale) in colitis rats. Only the mixture was able to modulate bacterial flora both in healthy rats and in TNBS-induced rat model of colitis. The mixture was also able to reduce the colonic damage score, iNOS expression as well as production of the TNFα and IL-1β and MPO activity, highlighting a synergism of papaya and kale association. Broccoli-supplemented diet have been reported to be effective in *mdr1a^−/−^ mice* (IBD mouse model) reducing colon inflammation and improving the intestine functionality, through modification of caecal microbiota composition and metabolism, and the colon morphology [100]. Moreover, *Lepidium virginicum* L., a plant widely used in traditional Mexican medicine as a remedy to treat gastrointestinal disorders, has been shown to significantly reduce colon inflammation, attenuating the clinical manifestations of colitis, immune cell infiltration, MPO activity, and some pro-inflammatory gene expression in DNBS-animal model of IBD [101]. The anti-inflammatory effects could be attributed to the phenolic acid and flavonoid content.

### 3.2. Effects of Brassicaceae-Derived Phytochemicals

The beneficial effects of Brassicaceae-derived phytochemicals have also been demonstrated. Many studies suggest that SFN is one of key anti-inflammatory components exerting many beneficial health properties [102,103,104,105]. SFN present in nanoparticles isolated from broccoli extracts (BDNs) seems to have a major contribution in the protection of mouse colitis by reducing the expression of inflammatory mediators and increasing the expression of nuclear factor (erythroid-derived 2)-like2 (Nrf2) dependent genes [106]**.**

Since heating inactivated the enzyme myrosinase involved in the formation of the bioactive compound SFN, Wang et al. analyzed the effect of cooking on anti-inflammation properties of edible Brassicaceae [107]. The authors demonstrated that although lightly-cooked broccoli contain a reduced myrosinase activity, they were still as effective as raw broccoli in reducing some injuries induced by DSS in mice, as the disease activity index (DAI), colon length, gut barrier permeability and colon lesion. Therefore, other broccoli compounds by themselves or in synergy with SFN may play a role in mitigating the colitis. Moreover, the anti-inflammatory effect induced by broccoli seems to be related to the removal of the Nrf2 inhibition of the IL-6 trans-signaling pathway, blocking the transition from acute to chronic inflammation [107].

As already explored, ethanol extract of the Brassicaceae *Wasabia Japonica* prevented the development of colitis in DSS mouse [92]. A further study demonstrated that *Wasabia japonica* anticolitic effects seems to be related to high content of allyl isothiocyanate (AITC), regulating tight junction proteins and mucin 2 (MUC2) via activation of ERK signaling [108].

In 2021, Lohning et al. reported that in vivo intraperitoneal injection of 6-(methylsulfinyl) hexyl Isothiocyanate (6-MITC) in DSS-induced murine model of colitis has potential anti-inflammatory alleviating some parameters, as weight loss, fecal blood, colon weight/length and levels of IL-6 and iNOS. Additionally, 6-MITC alleviates inflammation once more via inhibition of NF-kB signaling by inhibition of glycogen synthase kinase 3 beta (GSK-3β) [109]. Interestingly, 6-MITC is also a component of *Wasabia japonica*, once more supporting the potential anti-inflammatory activity of the Brassicaceae.

**Table 2:** In vivo effects of Brassicaceae on animal model of IBD.

Brassicaceae	Type of Extract	Dose	Animal Model	Reported Activity	Reference
**BRASSICACEAE EXTRACTS**					
*Brassica oleracea var. capitata rubra*	Ethanol Extract	5 mg/kg twice daily oral administration	TNBS/ DSS-mice	↓ inflammatory scores ↓ MPO activity, lipid peroxidation, ↓ IL-1β and TNF-α	[89]
*Raphanus sativus* L. *seeds*	Water Extract	100 mg/kg/d oral administration	TNBS/ DSS-model	↓ MPO, TNF-α, IL-1β, malondialdehyde production monocyte chemotactic protein-1, iNOS and intercellular adhesion molecule-1. ↓ NF-kB activities	[90]
*Broccoli*	BDNs	0.25 g/mouse/d oral administration	DSS-mice	↓ TNF-a, IL-17A, and IFN-γ, ↓ CD4^+^ T ↑ IL-10, activation: AMPK	[91]
*Wasabia Japonica roots*	Ethanol Extract	20–50–100 mg/kg/d oral administration	DSS-mice	↓ NF-kB signaling pathway recovery epithelial tight junctions	[92]
*Camelina sativa*	Defatted Seed Meal	1 g/kg/d oral administration	DNBS-rat	Relieves visceral hypersensitivity Prevents enteric neuron damage Modulates PPAR-α receptors	[94]
*Eruca sativa*	Water Extract	1 g/kg/d oral administration	DNBS-rat	Visceral anti-nociceptive effect Release of H_2_S ↑ Kv_7_ activity	[95]
*Maca * (*Lepidium meyenii*)	Crude Extract	100 mg/kg/d Oral gavage	DSS-mice	↓ Inflammatory scores ↓ MPO, TNF-α, IL-1β, IL-6. ↑ IL-10, intestinal tight junction	[98]
*Kale* (*Brassica oleracea*)	Diet	500 mg/kg/d (60%kale + 40% papaya) oral administration	TNBS-rat	↓ iNOS, TNF-α, IL-1β and MPO activity Modulation of bacterial flora	[99]
*Broccoli*	Supplemented Diet	Diet + 10%	*mdr1a*^−/−^ mice	Modulation of caecal microbiota composition and metabolism, ↑ colon morphology	[100]
*Raphanus sativus*	Ethanol Extract	40/70/100 mg/kg/d oral administration	DSS-mice	↓ COX-2, TNF-α, IL-1β, IL-6, PGE_2_ and MPO. ↓ IκB phosphorylation	[56]
*Lepidium* *virginicum*	Ethanol Extract	3–30–100 mg/kg/d Oral or Intraperitoneal injection	DNBS-rat	↓ inflammatory score, MPO activity, CXCL-1, ↓ TNF-α, and IL-1β	[101]
**BRASSICACAE-DERIVED PHYTOCHEMICALS**					
*Brassica-derived isothiocyanate sulforaphane*	SFN	25 mg/kg/d oral administration	DSS-mice	↓ inflammatory scores, ↑ Nrf2 dependent genes	[106]
*Broccoli*	SFN	supplemented diet preparation, either raw or lightly cooked	DSS-mice	↓ inflammatory scores, DAI ↓ IL-6, V-CAM1	[107]
*Wasabia japonica*	AITC	10 mg/kg/d oral administration	DSS-mice	↑ tight junction proteins, MUC-2	[108]
*Wasabia japonica*	6-MITC	10 mg/kg/d Oral or intraperitoneal injection	DSS-mice	↓ IL-6, iNOS, NF-kB and colon damage. GSK-3b inhibition	[109]

Abbreviations: AITC Allyl Isothiocyanate. AMPK: adenosine monophosphate-activated protein kinase. BDN nanoparticles isolated from extracts. CXCL-1: chemokine (C-X-C motif) ligand 1. COX-2: cyclooxygenase-2. DAI disease activity index. DNBS: 2,4 -dinitrobenzenesulfonic acid. DSS: dextran sodium sulfate. GSK-3b: glycogen synthase kinase 3 beta. H_2_S hydrogen sulphide. IFN-γ: Interferon gamma. IL: interleukin. iNOS: inducible nitric oxide synthase. *mdr1a*^−/−^*: mdr1a* knockout *mice.* 6-MITC: 6- (methylsulfinyl) hexyl Isothiocyanate. MPO: myeloperoxidase. MUC-2: mucin 2. NF-Κb: nuclear factor-κB. Nrf 2: nuclear factor (erythroid-derived 2)-like2. PGE-2: prostaglandin E2. PPAR: peroxisome proliferator-activated receptor alpha. SFN: sulforaphane. TNBS: 2,4,6-trinitrobenzenesulfonic acid. TNF-α: tumor necrosis factor alpha. VCAM-1 vascular cell adhesion molecule-1. ↓ decrease. ↑ increase.

## 4. Conclusions and Future Directions

The use of complementary and alternative therapies (as herbal preparations and dietary supplementations) in the treatment of IBD is increasing in recent years, due the side effects of conventional treatments and following the belief that complementary approaches are safe and natural [110].

In this context, Brassicaceae, already demonstrated to reduce cancer risk, cardiovascular disorders and diabetes [111,112,113], could represent a good support in IBD treatment, being an excellent sources of bioactive metabolites with high health-promoting benefits. Indeed, based on the various in vivo and in vitro studies, Brassica vegetables and other cruciferous foods have relevance in planning dietary recommendations for the prevention or relief of the pathological conditions due to chronic inflammatory diseases.

Among the phytochemicals present in Brassicaceae vegetables, calcium, fibers, vitamins A, C and a high variety of sulfur-containing and phenolic compounds, (hydroxycinnamic acids, and glucosinolates) can reduce inflammation. As discussed above, the anti-inflammatory effects are mainly the consequence of the reduction in the secretion of inflammatory mediators via modulation of the pro-inflammatory transcription factor, NF-κB signalling. However, additional efforts must be made regarding the discovery of new brassicaceous phytochemicals and eventual positive synergistic effects among them. Additionally, it is important to consider that the content of bioactive compounds in Brassicaceae vegetables is variable, depending on many factors as genotype, environmental, growth conditions and production practices and influenced by cooking methods [49,50,51,52,107].

Further studies should be addressed to investigate the differences among individuals, in terms as intestinal absorption, metabolism, age, gender and lifestyles, which can affect the beneficial proprieties. Moreover, although some patients suffering from Crohn’s disease can tolerate different Brassicaceae vegetables [114,115], they are in generally avoided as they are believed to worsen their symptoms. Therefore, it is mandatory to assess the health effects of single/a mixture of bioactive compounds to create easily available plant-based functional foods, dietary supplements, and products with prophylactic and/or therapeutic potential for IBD. We are aware that the in vitro and in vivo evidence reported in this review could encourage the screening and isolation of bioactive metabolites from Brassicaceae vegetables, with support of the ongoing development of high-throughput, efficient and reliable analytical approaches. In conclusion, all the research summarized in this review support the possibility that Brassicaceae compounds may prevent or treat inflammatory chronic diseases and indicate that further research should be oriented to develop Brassicaceae extracts enriched in bioactive compounds to be used in new anti-inflammatory therapies.

## Data Availability

No data was used for the research described in the article.

## References

[1] Hibi T., Ogata H. (2006). Novel Pathophysiological Concepts of Inflammatory Bowel Disease. J. Gastroenterol..

[2] Graham D.B., Xavier R.J. (2020). Pathway Paradigms Revealed from the Genetics of Inflammatory Bowel Disease. Nature.

[3] Zhang Y.Z., Li Y.Y. (2014). Inflammatory Bowel Disease: Pathogenesis. World J. Gastroenterol..

[4] Kobayashi T., Siegmund B., Le Berre C., Wei S.C., Ferrante M., Shen B., Bernstein C.N., Danese S., Peyrin-Biroulet L., Hibi T. (2020). Ulcerative Colitis. Nat. Rev. Dis. Prim..

[5] Qin X. (2013). Why Is Damage Limited to the Mucosa in Ulcerative Colitis but Transmural in Crohn’s Disease?. World J. Gastrointest. Pathophysiol..

[6] Danese S., Fiocchi C. (2006). Etiopathogenesis of Inflammatory Bowel Diseases. World J. Gastroenterol..

[7] Chang J.T. (2020). Pathophysiology of Inflammatory Bowel Diseases. N. Engl. J. Med..

[8] Strober W., Fuss I.J. (2011). Pro-Inflammatory Cytokines in the Pathogenesis of IBD. Gastroenterology.

[9] Liu T., Zhang L., Joo D., Sun S.C. (2017). NF-ΚB Signaling in Inflammation. Signal Transduct. Target. Ther..

[10] Hoesel B., Schmid J.A. (2013). The Complexity of NF-ΚB Signaling in Inflammation and Cancer. Mol. Cancer.

[11] Hayden M.S., Ghosh S. (2012). NF-ΚB, the First Quarter-Century: Remarkable Progress and Outstanding Questions. Genes Dev..

[12] Ng S.C., Shi H.Y., Hamidi N., Underwood F.E., Tang W., Benchimol E.I., Panaccione R., Ghosh S., Wu J.C.Y., Chan F.K.L. (2017). Worldwide Incidence and Prevalence of Inflammatory Bowel Disease in the 21st Century: A Systematic Review of Population-Based Studies. Lancet.

[13] Kassam Z., Belga S., Roifman I., Hirota S., Jijon H., Kaplan G.G., Ghosh S., Beck P.L. (2014). Inflammatory Bowel Disease Cause-Specific Mortality: A Primer for Clinicians. Inflamm. Bowel Dis..

[14] Kotze P.G., Steinwurz F., Francisconi C., Zaltman C., Pinheiro M., Salese L., Ponce de Leon D. (2020). Review of the Epidemiology and Burden of Ulcerative Colitis in Latin America. Therap. Adv. Gastroenterol..

[15] May D., Pan S., Crispin D.A., Lai K., Bronner M.P., Hogan J., Hockenbery D.M., McIntosh M., Brentnall T.A., Chen R. (2011). Investigating Neoplastic Progression of Ulcerative Colitis with Label-Free Comparative Proteomics. J. Proteome Res..

[16] Moreau J., Mas E. (2015). Drug Resistance in Inflammatory Bowel Diseases. Curr. Opin. Pharmacol..

[17] Coskun M., Vermeire S., Nielsen O.H. (2017). Novel Targeted Therapies for Inflammatory Bowel Disease. Trends Pharmacol. Sci..

[18] Na S.Y., Moon W. (2019). Perspectives on Current and Novel Treatments for Inflammatory Bowel Disease. Gut Liver.

[19] Polkinghorne I., Hamerli D., Cowan P., Duckworth J. (2005). Plant-Based Immunocontraceptive Control of Wildlife—“potentials, Limitations, and Possums”. Vaccine.

[20] Tabassum N., Hamdani M. (2014). Plants Used to Treat Skin Diseases. Pharmacogn. Rev..

[21] Duan L., Cheng S., Li L., Liu Y., Wang D., Liu G. (2021). Natural Anti-Inflammatory Compounds as Drug Candidates for Inflammatory Bowel Disease. Front. Pharmacol..

[22] Morsy M.A., Gupta S., Nair A.B., Venugopala K.N., Greish K., El-Daly M. (2019). Protective Effect of Spirulina Platensis Extract against Dextran-Sulfate-Sodium-Induced Ulcerative Colitis in Rats. Nutrients.

[23] Chang Y., Zhai L., Peng J., Wu H., Bian Z., Xiao H. (2021). Phytochemicals as Regulators of Th17/Treg Balance in Inflammatory Bowel Diseases. Biomed. Pharmacother..

[24] Zizzo M.G., Caldara G., Bellanca A., Nuzzo D., Di Carlo M., Scoglio S., Serio R. (2020). AphaMax^®^, an Aphanizomenon Flos-Aquae Aqueous Extract, Exerts Intestinal Protective Effects in Experimental Colitis in Rats. Nutrients.

[25] Abdel-Daim M.M., Farouk S.M., Madkour F.F., Azab S.S. (2015). Anti-Inflammatory and Immunomodulatory Effects of Spirulina Platensis in Comparison to Dunaliella Salina in Acetic Acid-Induced Rat Experimental Colitis. Immunopharmacol. Immunotoxicol..

[26] Al-Shehbaz I.A., Beilstein M.A., Kellogg E.A. (2006). Systematics and Phylogeny of the Brassicaceae (Cruciferae): An Overview. Plant Syst. Evol..

[27] Al-shehbaz I.A. (2011). Brassicaceae (Mustard Family). eLS.

[28] Ayadi J., Debouba M., Rahmani R., Bouajila J. (2022). Brassica Genus Seeds: A Review on Phytochemical Screening and Pharmacological Properties. Molecules.

[29] Raza A., Hafeez M.B., Zahra N., Shaukat K., Umbreen S., Tabassum J., Charagh S., Khan R.S.A., Hasanuzzaman M. (2020). The Plant Family Brassicaceae: Introduction, Biology, and Importance. The Plant Family Brassicaceae.

[30] Avato P., Argentieri M.P. (2015). Brassicaceae: A Rich Source of Health Improving Phytochemicals. Phytochem. Rev..

[31] Ramirez D., Abellán-Victorio A., Beretta V., Camargo A., Moreno D.A. (2020). Functional Ingredients From Brassicaceae Species: Overview and Perspectives. Int. J. Mol. Sci..

[32] Jahangir M., Kim H.K., Choi Y.H., Verpoorte R. (2009). Health-Affecting Compounds in Brassicaceae. Compr. Rev. Food Sci. Food Saf..

[33] Wang L.I., Giovannucci E.L., Hunter D., Neuberg D., Su L., Christiani D.C. (2004). Dietary Intake of Cruciferous Vegetables, Glutathione S-Transferase (GST) Polymorphisms and Lung Cancer Risk in a Caucasian Population. Cancer Causes Control.

[34] Jung U.J., Baek N.I., Chung H.G., Bang M.H., Jeong T.S., Tae Lee K., Kang Y.J., Lee M.K., Kim H.J., Yeo J. (2008). Effects of the Ethanol Extract of the Roots of Brassica Rapa on Glucose and Lipid Metabolism in C57BL/KsJ-Db/Db Mice. Clin. Nutr..

[35] An S., Han J.-I., Kim M.J., Park J.S., Han J.M., Baek N.I., Chung H.G., Choi M.S., Lee K.T., Jeong T.S. (2010). Ethanolic Extracts of Brassica Campestris Spp. Rapa Roots Prevent High-Fat Diet-Induced Obesity via Beta (3)-Adrenergic Regulation of White Adipocyte Lipolytic Activity. J. Med. Food.

[36] Wiczkowski W., Szawara-Nowak D., Topolska J. (2013). Red Cabbage Anthocyanins: Profile, Isolation, Identification, and Antioxidant Activity. Food Res. Int..

[37] Romani A., Vignolini P., Isolani L., Ieri F., Heimler D. (2006). HPLC-DAD/MS Characterization of Flavonoids and Hydroxycinnamic Derivatives in Turnip Tops (*Brassica rapa* L. Subsp. *Sylvestris* L.). J. Agric. Food Chem..

[38] Šamec D., Pavlović I., Salopek-Sondi B. (2017). White Cabbage (*Brassica oleracea* Var. *Capitata* f. Alba): Botanical, Phytochemical and Pharmacological Overview. Phytochem. Rev..

[39] Schonhof I., Krumbein A., Brückner B. (2004). Genotypic Effects on Glucosinolates and Sensory Properties of Broccoli and Cauliflower. Nahrung.

[40] Ares A.M., Nozal M.J., Bernal J. (2013). Extraction, Chemical Characterization and Biological Activity Determination of Broccoli Health Promoting Compounds. J. Chromatogr. A.

[41] Choe U., Li Y., Gao B., Yu L., Wang T.T.Y., Sun J., Chen P., Liu J., Yu L. (2018). Chemical Compositions of Cold-Pressed Broccoli, Carrot, and Cucumber Seed Flours and Their in vitro Gut Microbiota Modulatory, Anti-Inflammatory, and Free Radical Scavenging Properties. J. Agric. Food Chem..

[42] Jensen J., Styrishave B., Gimsing A.L., Hansen H.C.B. (2010). The Toxic Effects of Benzyl Glucosinolate and Its Hydrolysis Product, the Biofumigant Benzyl Isothiocyanate, to Folsomia Fimetaria. Environ. Toxicol. Chem..

[43] Smolinska U., Morra M.J., Knudsen G.R., Brown P.D. (1997). Toxicity of Glucosinolate Degradation Products from Brassica Napus Seed Meal Toward Aphanomyces Euteiches f. Sp. Pisi. Phytopathology.

[44] Holst B., Fenwick G.R. (2003). Glucosinolates. Encycl. Food Sci. Nutr..

[45] Quirante-Moya S., García-Ibañez P., Quirante-Moya F., Villaño D., Moreno D.A. (2020). The Role of Brassica Bioactives on Human Health: Are We Studying It the Right Way?. Molecules.

[46] Prieto M.A., López C.J., Simal-Gandara J. (2019). Glucosinolates: Molecular Structure, Breakdown, Genetic, Bioavailability, Properties and Healthy and Adverse Effects. Adv. Food Nutr. Res..

[47] Kandala P.K., Wright S.E., Srivastava S.K. (2012). Blocking Epidermal Growth Factor Receptor Activation by 3,3′-Diindolylmethane Suppresses Ovarian Tumor Growth In vitro and In vivo. J. Pharmacol. Exp. Ther..

[48] Houghton C.A. (2019). Sulforaphane: Its “Coming of Age” as a Clinically Relevant Nutraceutical in the Prevention and Treatment of Chronic Disease. Oxid. Med. Cell. Longev..

[49] Ahuja I., de Vos R.C.H., Bones A.M., Hall R.D. (2010). Plant Molecular Stress Responses Face Climate Change. Trends Plant Sci..

[50] Lo Scalzo R., Bianchi G., Genna A., Summa C. (2007). Antioxidant Properties and Lipidic Profile as Quality Indexes of Cauliflower (*Brassica oleracea* L. Var. *Botrytis*) in Relation to Harvest Time. Food Chem..

[51] Schonhof I., Kläring H.P., Krumbein A., Claußen W., Schreiner M. (2007). Effect of Temperature Increase under Low Radiation Conditions on Phytochemicals and Ascorbic Acid in Greenhouse Grown Broccoli. Agric. Ecosyst. Environ..

[52] Valente Pereira F.M., Rosa E., Fahey J.W., Stephenson K.K., Carvalho R., Aires A. (2002). Influence of Temperature and Ontogeny on the Levels of Glucosinolates in Broccoli (*Brassica oleracea* Var. *Italica*) Sprouts and Their Effect on the Induction of Mammalian Phase 2 Enzymes. J. Agric. Food Chem..

[53] Scialabba A., Salvini L., Faqi A.S., Bellani L.M. (2010). Tocopherol, Fatty Acid and Phytosterol Content in Seeds of Nine Wild Taxa of Sicilian Brassica (Cruciferae). Plant Biosyst..

[54] Serhan C.N. (2011). The Resolution of Inflammation: The Devil in the Flask and in the Details. FASEB J..

[55] Michael S., Kelber O., Hauschildt S., Spanel-Borowski K., Nieber K. (2009). Inhibition of Inflammation-Induced Alterations in Rat Small Intestine by the Herbal Preparations STW 5 and STW 6. Phytomedicine.

[56] Kim G., Jang M., Hwang I., Cho J., Kim S. (2021). Radish Sprout Alleviates DSS-Induced Colitis via Regulation of NF-KB Signaling Pathway and Modifying Gut Microbiota. Biomed. Pharmacother..

[57] Hwang J.H., Lim S. (2014). Bin Antioxidant and Anti-Inflammatory Activities of Broccoli Florets in LPS-Stimulated RAW 264.7 Cells. Prev. Nutr. Food Sci..

[58] Rokayya S., Li C.J., Zhao Y., Li Y., Sun C.H. (2013). Cabbage (*Brassica oleracea* L. Var. *Capitata*) Phytochemicals with Antioxidant and Anti-Inflammatory Potential. Asian Pac. J. Cancer Prev..

[59] Olszewska M.A., Granica S., Kolodziejczyk-Czepas J., Magiera A., Czerwinska M.E., Nowak P., Rutkowska M., Wasinski P., Owczarek A. (2020). Variability of Sinapic Acid Derivatives during Germination and Their Contribution to Antioxidant and Anti-Inflammatory Effects of Broccoli Sprouts on Human Plasma and Human Peripheral Blood Mononuclear Cells. Food Funct..

[60] Ruiz-Alcaraz A.J., Martínez-Sánchez M.A., García-Peñarrubia P., Martinez-Esparza M., Ramos-Molina B., Moreno D.A. (2022). Analysis of the Anti-Inflammatory Potential of Brassica Bioactive Compounds in a Human Macrophage-like Cell Model Derived from HL-60 Cells. Biomed. Pharmacother..

[61] Lin J.Y., Li C.Y., Hwang I.F. (2008). Characterisation of the Pigment Components in Red Cabbage (*Brassica oleracea* L. Var.) Juice and Their Anti-Inflammatory Effects on LPS-Stimulated Murine Splenocytes. Food Chem..

[62] Shin J.S., Noh Y.S., Lee Y.S., Cho Y.W., Baek N.I., Choi M.S., Jeong T.S., Kang E., Chung H.G., Lee K.T. (2011). Arvelexin from Brassica Rapa Suppresses NF-ΚB-Regulated pro-Inflammatory Gene Expression by Inhibiting Activation of IκB Kinase. Br. J. Pharmacol..

[63] Guzik T.J., Korbut R., Adamek-Guzik T. (2003). Nitric Oxide and Superoxide in Inflammation and Immune Regulation. J. Physiol. Pharmacol..

[64] Oeckinghaus A., Ghosh S. (2009). The NF-KappaB Family of Transcription Factors and Its Regulation. Cold Spring Harb. Perspect. Biol..

[65] Geum N.G., Yeo J.H., Yu J.H., Choi M.Y., Lee J.W., Baek J.K., Jeong J.B. (2021). In vitro Immunostimulatory Activity of Bok Choy (Brassica Campestris Var. Chinensis) Sprouts in RAW264.7 Macrophage Cells. Korean J. Plant Resour..

[66] Cho S.H., Kim S.R., Jeong M.S., Choi M., Park S.J., Kim K.N. (2021). Protective Effect of *Brassica napus* L. Hydrosols against Inflammation Response in RAW 264.7 Cells. Chin. J. Integr. Med..

[67] Kuntz S., Kunz C. (2014). Extracts from *Brassica oleracea* L. Convar. Acephala Var. Sabellica Inhibit TNF-α Stimulated Neutrophil Adhesion in vitro under Flow Conditions. Food Funct..

[68] Rose P., Yen K.W., Choon N.O., Whiteman M. (2005). Beta-Phenylethyl and 8-Methylsulphinyloctyl Isothiocyanates, Constituents of Watercress, Suppress LPS Induced Production of Nitric Oxide and Prostaglandin E2 in RAW 264.7 Macrophages. Nitric Oxide Biol. Chem..

[69] Heiss E., Herhaus C., Klimo K., Bartsch H., Gerhäuser C. (2001). Nuclear Factor Kappa B Is a Molecular Target for Sulforaphane-Mediated Anti-Inflammatory Mechanisms. J. Biol. Chem..

[70] Bachiega P., Salgado J.M., De Carvalho J.E., Ruiz A.L.T.G., Schwarz K., Tezotto T., Morzelle M.C. (2016). Antioxidant and Antiproliferative Activities in Different Maturation Stages of Broccoli (*Brassica oleracea Italica*) Biofortified with Selenium. Food Chem..

[71] Pia X.L., Im H.Y.I., Yokozawa T., Lees Y.A., Piao X.S., Cho E.J. (2005). Protective effects of broccoli (Brassica oleracea) and its active components against radical-induced oxidative damage. J. Nutr. Sci. Vitaminol..

[72] Ayaz F.A., Hayirlioglu-Ayaz S., Alpay-Karaoglu S., Grúz J., Valentová K., Ulrichová J., Strnad M. (2008). Phenolic Acid Contents of Kale (*Brassica oleraceae* L. Var. *Acephala* DC.) Extracts and Their Antioxidant and Antibacterial Activities. Food Chem..

[73] Wu H., Zhu J., Yang L., Wang R., Wang C. (2015). Ultrasonic-Assisted Enzymatic Extraction of Phenolics from Broccoli (*Brassica oleracea* L. Var. *Italica*) Inflorescences and Evaluation of Antioxidant Activity in vitro. Food Sci. Technol. Int..

[74] Le T.N., Chiu C., Hsieh P. (2020). Bioactive compounds and bioactivities of *Brassica oleracea* L. var. *Italica* sprouts and microgreens: An updated overview from a nutraceutical perspective. Plants.

[75] Bhatt S., Singh B., Gupta M. (2020). Antioxidant and Prebiotic Potential of *Murraya koenigii* and *Brassica oleracea* Var. *Botrytis leaves* as Food Ingredient. J. Agric. Food Res..

[76] Picchi V., Lo Scalzo R., Tava A., Doria F., Argento S., Toscano S., Treccarichi S., Branca F. (2020). Phytochemical Characterization and In vitro Antioxidant Properties of Four Brassica Wild Species from Italy. Molecules.

[77] de Oliveira I.R.N., Roque J.V., Maia M.P., Stringheta P.C., Teófilo R.F. (2018). New Strategy for Determination of Anthocyanins, Polyphenols and Antioxidant Capacity of *Brassica oleracea* Liquid Extract Using Infrared Spectroscopies and Multivariate Regression. Spectrochim. Acta Part A Mol. Biomol. Spectrosc..

[78] Chew S.C. (2020). Cold-Pressed Rapeseed (Brassica Napus) Oil: Chemistry and Functionality. Food Res. Int..

[79] Kaulmann A., Jonville M.C., Schneider Y.J., Hoffmann L., Bohn T. (2014). Carotenoids, Polyphenols and Micronutrient Profiles of Brassica Oleraceae and Plum Varieties and Their Contribution to Measures of Total Antioxidant Capacity. Food Chem..

[80] Chang J., Wang M., Jian Y., Zhang F., Zhu J., Wang Q., Sun B. (2019). Health-Promoting Phytochemicals and Antioxidant Capacity in Different Organs from Six Varieties of Chinese Kale. Sci. Rep..

[81] Chaudhary A., Choudhary S., Sharma U., Vig A.P., Singh B., Arora S. (2018). Purple Head Broccoli (*Brassica oleracea* L. Var. *Italica* Plenck), a Functional Food Crop for Antioxidant and Anticancer Potential. J. Food Sci. Technol..

[82] Usami A., Motooka R., Takagi A., Nakahashi H., Okuno Y., Miyazawa M. (2014). Chemical Composition, Aroma Evaluation, and Oxygen Radical Absorbance Capacity of Volatile Oil Extracted from Brassica Rapa Cv. “Yukina” Used in Japanese Traditional Food. J. Oleo Sci..

[83] Kurilich A.C., Jeffery E.H., Juvik J.A., Wallig M.A., Klein B.P. (2002). Antioxidant Capacity of Different Broccoli (Brassica Oleracea) Genotypes Using the Oxygen Radical Absorbance Capacity (ORAC) Assay. J. Agric. Food Chem..

[84] Eun J.C., Lee Y.A., Hye H.Y., Yokozawa T. (2006). Protective Effects of Broccoli (*Brassica oleracea*) against Oxidative Damage in vitro and in vivo. J. Nutr. Sci. Vitaminol..

[85] Kaulmann A., Planchon S., Renaut J., Schneider Y.J., Hoffmann L., Bohn T. (2016). Proteomic Response of Inflammatory Stimulated Intestinal Epithelial Cells to: In vitro Digested Plums and Cabbages Rich in Carotenoids and Polyphenols. Food Funct..

[86] Goyal N., Rana A., Ahlawat A., Bijjem K.R.V., Kumar P. (2014). Animal Models of Inflammatory Bowel Disease: A Review. Inflammopharmacology.

[87] Baydi Z., Limami Y., Khalki L., Zaid N., Naya A., Mtairag E.M., Oudghiri M., Zaid Y. (2021). An Update of Research Animal Models of Inflammatory Bowel Disease. Sci. World J..

[88] Ghattamaneni N.K.R., Panchal S.K., Brown L. (2018). Nutraceuticals in Rodent Models as Potential Treatments for Human Inflammatory Bowel Disease. Pharmacol. Res..

[89] Zielińska M., Lewandowska U., Podsedek A., Cygankiewicz A.I., Jacenik D., Sałaga M., Kordek R., Krajewska W.M., Fichna J. (2015). Orally Available Extract from *Brassica oleracea* Var. Capitata Rubra Attenuates Experimental Colitis in Mouse Models of Inflammatory Bowel Diseases. J. Funct. Foods.

[90] Choi K.C., Cho S.W., Kook S.H., Chun S.R., Bhattarai G., Poudel S.B., Kim M.K., Lee K.Y., Lee J.C. (2016). Intestinal Anti-Inflammatory Activity of the Seeds of *Raphanus sativus* L. in Experimental Ulcerative Colitis Models. J. Ethnopharmacol..

[91] Deng Z., Rong Y., Teng Y., Mu J., Zhuang X., Tseng M., Samykutty A., Zhang L., Yan J., Miller D. (2017). Broccoli-Derived Nanoparticle Inhibits Mouse Colitis by Activating Dendritic Cell AMP-Activated Protein Kinase. Mol. Ther..

[92] Kang J.H., Choi S., Jang J.E., Ramalingam P., Ko Y.T., Kim S.Y., Oh S.H. (2017). Wasabia Japonica Is a Potential Functional Food to Prevent Colitis via Inhibiting the NF-ΚB Signaling Pathway. Food Funct..

[93] Chaturvedi S., Bhattacharya A., Khare S.K., Kaushik G., Hussain C. (2019). Camelina Sativa: An Emerging Biofuel Crop. Handbook of Environmental Materials Management.

[94] Lucarini E., Micheli L., Pagnotta E., Toti A., Ferrara V., Ciampi C., Margiotta F., Martelli A., Testai L., Calderone V. (2022). The Efficacy of Camelina Sativa Defatted Seed Meal against Colitis-Induced Persistent Visceral Hypersensitivity: The Relevance of PPAR α Receptor Activation in Pain Relief. Nutrients.

[95] Lucarini E., Micheli L., Pagnotta E., Matteo R., Parisio C., Toti A., Ferrara V., Ciampi C., Martelli A., Testai L. (2022). Beneficial Effects of Eruca Sativa Defatted Seed Meal on Visceral Pain and Intestinal Damage Resulting from Colitis in Rats. Foods.

[96] Zha S., Zhao Q., Chen J., Wang L., Zhang G., Zhang H., Zhao B. (2014). Extraction, Purification and Antioxidant Activities of the Polysaccharides from Maca (*Lepidium meyenii*). Carbohydr. Polym..

[97] Chang Y., Lu W., Chu Y., Yan J., Wang S., Xu H., Ma H., Ma J. (2020). Extraction of Polysaccharides from Maca: Characterization and Immunoregulatory Effects on CD4+ T Cells. Int. J. Biol. Macromol..

[98] Zha R., Ge E., Guo L., Gao Q., Lin Q., Zhou W., Jin X., Xie W., Yin H., Liu T. (2021). A Newly Identified Polyunsaturated Macamide Alleviates Dextran Sulfate Sodium-Induced Colitis in Mice. Fitoterapia.

[99] Lima de Albuquerque C., Comalada M., Camuesco D., Rodríguez-Cabezas M.E., Luiz-Ferreira A., Nieto A., Monteiro de Souza Brito A.R., Zarzuelo A., Gálvez J. (2010). Effect of Kale and Papaya Supplementation in Colitis Induced by Trinitrobenzenesulfonic Acid in the Rat. e-SPEN.

[100] Paturi G., Mandimika T., Butts C.A., Zhu S., Roy N.C., McNabb W.C., Ansell J. (2012). Influence of Dietary Blueberry and Broccoli on Cecal Microbiota Activity and Colon Morphology in Mdr1a -/- Mice, a Model of Inflammatory Bowel Diseases. Nutrition.

[101] Cruz-Muñoz J.R., Barrios-García T., Valdez-Morales E.E., Durán-Vazquez M.F., Méndez-Rodríguez K.B., Barajas-Espinosa A., Ochoa-Cortes F., Martínez-Saldaña M.C., Gómez-Aguirre Y.A., Alba R.G. (2022). Ethanolic Extract from *Lepidium virginicum* L. Ameliorates DNBS-Induced Colitis in Rats. J. Ethnopharmacol..

[102] Al-Bakheit A., Abu-Qatouseh L. (2020). Sulforaphane from Broccoli Attenuates Inflammatory Hepcidin by Reducing IL-6 Secretion in Human HepG2 Cells. J. Funct. Foods.

[103] Miekus N., Marszałek K., Podlacha M., Iqbal A., Puchalski C., Swiergiel A.H. (2020). Health Benefits of Plant-Derived Sulfur Compounds, Glucosinolates, and Organosulfur Compounds. Molecules.

[104] Mithen R. (2007). Sulphur-Containing Compounds. Plant Secondary Metabolites: Occurrence, Structure and Role in the Human Diet.

[105] Gasper A.V., Al-Janobi A., Smith J.A., Bacon J.R., Fortun P., Atherton C., Taylor M.A., Hawkey C.J., Barrett D.A., Mithen R.F. (2005). Glutathione S-Transferase M1 Polymorphism and Metabolism of Sulforaphane from Standard and High-Glucosinolate Broccoli. Am. J. Clin. Nutr..

[106] Wagner A.E., Will O., Sturm C., Lipinski S., Rosenstiel P., Rimbach G. (2013). DSS-Induced Acute Colitis in C57BL/6 Mice Is Mitigated by Sulforaphane Pre-Treatment. J. Nutr. Biochem..

[107] Wang Y., Jeffery E.H., Miller M.J., Wallig M.A., Wu Y. (2018). Lightly Cooked Broccoli Is as Effective as Raw Broccoli in Mitigating Dextran Sulfate Sodium-Induced Colitis in Mice. Nutrients.

[108] Kim M.W., Choi S., Kim S.Y., Yoon Y.S., Kang J.H., Oh S.H. (2018). Allyl Isothiocyanate Ameliorates Dextran Sodium Sulfate-Induced Colitis in Mouse by Enhancing Tight Junction and Mucin Expression. Int. J. Mol. Sci..

[109] Lohning A., Kidachi Y., Kamiie K., Sasaki K., Ryoyama K., Yamaguchi H. (2021). 6- (Methylsulfinyl) Hexyl Isothiocyanate (6-MITC) from Wasabia Japonica Alleviates Inflammatory Bowel Disease (IBD) by Potential Inhibition of Glycogen Synthase Kinase 3 Beta (GSK-3β). Eur. J. Med. Chem..

[110] Lin S.C., Cheifetz A.S. (2018). The Use of Complementary and Alternative Medicine in Patients With Inflammatory Bowel Disease. Gastroenterol. Hepatol. (N. Y)..

[111] Kristal A.R., Lampe J.W. (2002). Brassica Vegetables and Prostate Cancer Risk: A Review of the Epidemiological Evidence. Nutr. Cancer.

[112] Cornelis M.C., El-Sohemy A., Campos H. (2007). GSTT1 Genotype Modifies the Association between Cruciferous Vegetable Intake and the Risk of Myocardial Infarction. Am. J. Clin. Nutr..

[113] Chen G.C., Koh W.P., Yuan J.M., Qin L.Q., Van Dam R.M. (2018). Green Leafy and Cruciferous Vegetable Consumption and Risk of Type 2 Diabetes: Results from the Singapore Chinese Health Study and Meta-Analysis. Br. J. Nutr..

[114] Campbell B., Han D.Y., Triggs C.M., Fraser A.G., Ferguson L.R. (2012). Brassicaceae: Nutrient Analysis and Investigation of Tolerability in People with Crohn’s Disease in a New Zealand Study. Funct. Foods Heal. Dis..

[115] Laing B., Han D.Y., Ferguson L.R. (2013). Candidate Genes Involved in Beneficial or Adverse Responses to Commonly Eaten Brassica Vegetables in a New Zealand Crohn’s Disease Cohort. Nutrients.

